# Development of a Brief Caregiver-centric Screening Tool to Identify Risk of Depression among Caregivers of Hospitalized Older Adults

**DOI:** 10.1007/s12603-019-1197-7

**Published:** 2019-04-25

**Authors:** Ee-Yuee Chan, Z.-X. Lim, Y.Y. Ding, Y.H. Chan, W.S. Lim

**Affiliations:** 1 11 Jalan Tan Tock Seng, Nursing Service, Annex 1, Tan Tock Seng Hospital, 308433, Singapore, Singapore; 2 Alice Lee Centre of Nursing Studies, National University of Singapore, Singapore, Singapore; 3 Department of Geriatric Medicine, Institute of Geriatric and Active Aging, Tan Tock Seng Hospital, Singapore, Singapore; 4 Biostatistics Unit Yong Loo Lin School of Medicine, National University of Singapore National University Health System, Singapore, Singapore

**Keywords:** Screening tool, caregiver, depression, burden, mastery, quality of life, older adults

## Abstract

**Objectives:**

Caregivers of hospitalized older adults experience elevated levels of stress and are at risk of poor health outcomes. There is a lack of screening tools based on self-reported caregiver variables incorporating both protective and risk factors, for early identification of at-risk caregivers. This study reports the development of a caregiver-centric screening tool to identify risk of depression at admission and predicts 3-month risk of depression and quality of life amongst caregivers of older adults with an unplanned admission.

**Design, Setting and Participants:**

This prospective cohort study was conducted in the medical wards of a tertiary-care hospital from July 2015 to May 2017. We recruited family caregivers of patients aged 65 years and above who fulfilled the following criteria: a) unplanned admission, b) not residing in nursing homes; and c) requiring assistance in activities of daily living.

**Measurements:**

We examined 11 candidate caregiver variables (mastery, burden and nine demographic variables). Risk of depression (score ≥8 on Hospital Anxiety and Depression Scale (HADS-D) depression subscale) was the primary outcome, and was assessed during the index admission. Logistic regression models were used to identify risk factors and risk scores (weights). The total risk scores were then stratified into three risk levels. Predictive validity of the screening tool was assessed using 3-months post-discharge risk of depression and health-related quality of life (HRQoL).

**Results:**

The study included 274 caregiver-patient dyads. The mean (SD) age of the caregivers was 59 (10) years with 33.6% caregivers screening positive for risk of depression. The final model comprised three caregiver variables: mastery, burden and education. The total risk scores ranged from 0 to 6 and showed good discrimination (AUC:0.82, 95% CI: 0.77 to 0.87). Caregivers were classified into low-risk (0–1 points), intermediate-risk (2–4 points), and high-risk (5–6 points) groups, with corresponding rates of risk of depression (HADS-D≥8) of 10.7%, 44.6% and 73.3%, during admission. Relative risk rates of the intermediate- and high-risk group using the low-risk group as reference were 4.16 and 6.84 respectively. At 3-months post-discharge, the rates of caregivers at risk of depression or having poor HRQoL also increased corresponding to the three risk levels as per baseline, supporting the predictive validity of the tool.

**Conclusions/Implications:**

The caregiver-centric tool is a novel, practical, self-administered, relatively brief caregiver-centric instrument that can be used for rapid screening and stratification of caregivers at risk of depression. Uniquely, the tool comprised of assessment of protective factor (mastery) in addition to risk factors to provide a holistic assessment of the caregiver. It can be incorporated as part of older adults' admission evaluation so that prompt intervention can be rendered to their at-risk caregivers.

## Background

Globally, the number of individuals aged 60 and above will triple from 524 million in 2010 to 1.5 billion in 2050 (1). Accompanying this rise will be the number of older adults admitted into hospitals, often for acute exacerbations of pre-existing chronic diseases. These older adults requiring hospitalisation tend to be frail, suffer from more severe diseases and disabilities (2, 3). Without adequate support, their family caregivers can be overwhelmed by stressors from the caregiving role (4, 5, 6).

When family caregivers are overwhelmed by the caregiving role, they and in turn their care-recipients suffer from negative outcomes including poorer quality of life. Studies in both Western and Asian populations have shown that caregivers report higher levels of depression and poorer quality of life as compared to non-caregivers (5, 6, 7). These negative outcomes are further exacerbated when older adults have concomitant behavioural problems from dementia or have daily functional limitations (6, 8). Notably, depression in caregivers is an important indicator of other caregiving related issues. A study on dementia caregivers showed that symptoms of depression in caregivers were significantly associated with unmet caregiving needs such as dementia education, mental health care and medical care (9). Caregivers who are experiencing high stress also tend to provide poorer care and have a higher tendency to place their care-recipients in nursing homes (10, 11). These highlight the importance of early identification of at-risk caregivers for target interventions.

In caregiver research, a number of instruments have been developed to assess the burden of caregiving. One of the most consistently used in dementia caregiving research is the Zarit Burden Interview (ZBI) which has 22 items and assesses multiple domains, including health, social, economic, emotional, and family support (12). It was initially developed for caregivers of dementia patients and has since been validated and used in diverse populations, including older adults. Another tool, the caregiver strain index, assesses strain related to care provision, and was designed for family caregivers of older adults in the community (13). Professional judgement is required to interpret the level of caregiver strain as total scores are not categorised to different strain levels. In addition, there are scales which assess specific stressors such as the Neuropsychiatric Inventory–Questionnaire which assesses neuropsychiatric symptoms (14); or were developed for a specific population, such as the Weitzner's Quality-of-Life-Index-Cancer which was developed for cancer caregiver-patient dyads (15).

Despite the availability of assessment tools and effective interventions (16), few hospital settings routinely screen for risk of depression in caregivers of older adults (17). This could be attributed to the lack of suitable screening tools in these settings. Firstly, most available tools are time consuming, or require the presence of a trained interviewer to administer due to the complexity or context of the assessment (12, 18). Additionally, the translation to clinical utility is limited by the absence of specific cut-off scores for risk stratification. This makes it challenging to judge which group of caregivers are at higher risk of negative health outcomes (13, 19). Available tools may also have been developed for specific patient population, and may not be appropriate for hospitalized older adults. Importantly, protective factors, such as the psychological status and the ability to cope with the demands of caregiving are often not included, limiting a fuller assessment of the caregivers (12, 13, 19).

Acknowledging the challenges of acute care settings, we developed a brief screening tool based on self-reported caregiver variables to minimise respondent burden on the healthcare workers to encourage its adoption. As the tool is meant to be clinically interpretable, caregivers will be stratified into different risk levels according to their scores and the users can provide the appropriate caregiver support according to these risk levels. Such risk stratification is also necessary when there are scarce healthcare resources so that more help can be prioritised for severely distressed caregivers. We focused on caregivers of hospitalised older adults as these older adults constitute a frail population with higher care needs. Nonetheless, we expect the tool to be applicable in other settings as the tool was not developed for a specific underlying health condition.

We adopted a stress-appraisal framework (20, 21) that incorporated the seminal caregiving appraisal model (21) and the stress process model (22). Guided by the stress-appraisal model, candidate caregiver variables included background and contextual factors (age, gender, marital status, relationship to care-recipient, working status, education and housing type, living arrangement and absence of domestic helper), caregiver burden (secondary appraisal of the caregiving situation manifested as overload or burden), and mastery (protective factor that moderates the effect of stressors), all of which act in concert to influence outcomes (depression and quality of life).

A key component of the tool would be the assessment of protective factor, in addition to risk factors to provide a more holistic assessment of the caregiver. The construct chosen to represent protective factor was the caregivers' sense of mastery. Mastery, defined as the extent to which one regards one's life as being under their own control, is conceptualised as an overarching self-concept in the stress process model (22). The mastery construct is validated (23), malleable (24), caregiver specific and assesses a general sentiment of the caregiving situation, making it ideal for caregiver screening. According to Pearlin, mastery can reduce the impact of stress either via people with high mastery perceiving stressors in a less menacing way, or with it acting as a self-fulfilling prophecy (25). Recent research found that lower levels of mastery was associated with higher levels of burden, which eventually lead to higher levels of depression (26, 27). Mastery was also shown to attenuate the effects of stress on health across time, while low mastery was associated with depressive symptoms and poor health (28).

This provided the impetus for our current study, in which we aimed to develop a brief caregiver-centric practical risk score that identifies risk of depression at admission and predicts 3-month risk of depression and quality of life amongst caregivers of older adults with an unplanned admission. This risk score can then be applied as a routine screening tool in hospital settings to identify and stratify at-risk caregivers for interventions to be targeted.

## Methods

### Study setting and Participants

This single site prospective cohort study was conducted between July 2015 to May 2017 at the inpatient wards of a 1300-bedded hospital in Singapore. Of the 367 caregiver-patient dyad screened, 93 (25%) rejected participation. The final sample yielded 274 caregiver-patient dyads. We defined family caregivers as unpaid family members with the responsibility of decision making and caring for their loved ones. The inclusion criteria were family caregivers of patients aged 65 years and above who fulfilled the following criteria: a) unplanned admission, b) not residing in nursing homes; and c) requiring assistance in activities of daily living. We excluded caregivers of patients who were residents of nursing homes as these caregivers were not providing caregiving duties to their loved ones.

Ethics approval was obtained from the National Healthcare Group domain specific review board. Written informed consent was obtained from participating caregivers and verbal consent was obtained from family members who were cognitively intact.

### Study Procedure

Potential participants were identified through screening of the daily hospitalization lists of patients admitted to the Geriatrics and General Medical wards. Two trained interviewers administered the questionnaires at two time-points: upon admission and at 3-months post-discharge. During the first time point, the questionnaire was administered once the patients were deemed stable by the medical team, to prevent attendant stress associated with hospitalization. The caregivers were asked to frame their responses based on the time-frame of two weeks prior to the index hospitalization to establish their status at home immediately prior to hospitalization, when they were fully responsible for caregiving duties. At 3-months post-discharge, caregivers completed a follow-up questionnaire. For non-English speaking participants a Chinese version of the questionnaire was used (29, 30, 31, 32). The questionnaire took about 15 to 20 minutes to complete.

### Risk Factors

Candidate risk factors were identified through theoretical relevancy or were previously identified risk factors for poor health outcomes. As the screening tool was developed to allow rapid self-administration by the caregivers to increase its clinical utility, we also only considered self-reported caregiver variables and excluded clinical variables. Cut-off points for the candidate risk factors were chosen based on previous studies and data distributions.

Caregiver demographic risk factors included older age (≥65 years) (6), marital status (single) (5), relationship to care-recipient (spouse) (33, 34), working status (33) and sex (female) (34, 35). Social economic status indicators included low education (secondary school or lower) (34, 36) and housing type (public housing). Indicators of care demands such as living with care-recipient (33) and the absence of a domestic helper were also included. Two psychological risk factors measured were mastery and caregiver burden.

The measurements chosen for the constructs of mastery and burden, and for the outcomes of risk of depression and HRQoL, were based on the measurements being widely used in caregiver populations and on them having been validated.

### Mastery

Mastery was assessed using the 7-item scale developed by Pearlin and Schooler (37). This measure was chosen as it is widely used to measure global mastery in caregivers. The scale has good internal consistency (alpha 0.75 to 0.79). Items were scored on a 4-point scale. The scores were recoded such that each item ranged from 0 to 3, with a higher total score representing lower levels of mastery (0 to 21). For this study, low mastery was defined as a score of ≥10 (38), which corresponded to the tertile cutoff in our study population.

### Caregiving Burden

Perceived burden was assessed using the ZBI (12). The ZBI is one of the most widely used scales to assess burden experienced by caregivers and it has been validated in multiple settings. This study uses the 4-item screening version from the original 22-item ZBI (39). Each item was scored on a 5-point scale (0 to 4). Previous studies have showed that correlations between the screening version and the full version varied from 0.83 to 0.93 (39). A higher score indicates higher burden, and the total score ranges from 0 to 16. For this study, high burden was defined as a score of ≥8 (39).

### Outcome Variables

#### Risk of depression

Risk of depression as measured by the depression subscale of the Hospital Anxiety and Depression Scale (HADS-D) (40), was the primary outcome in this study. The HADS-D is a self-assessment tool that assesses the construct of depression in the setting of medical practice, largely (not entirely) from the reflections of the state of the loss of pleasure response (anhedonia) (40). The HADS-D has performed well in assessing severity and screening of depression in both somatic and psychiatric cases. It has been shown to be valid in the hospital and community settings (40), and has been previously used on family caregivers (41, 42). Each of the seven items are scored from 0 to 3, and the total score ranges from 0 to 21. HADS-D provides cut-off scores for risk stratifications. For this study, we used the HADS-D cutoff score of ≥8 to define risk of depression (43).

#### Health-related Quality of Life

Health related quality of life (HRQoL) was measured using the Short Form-12 version 2 (SF-12). The SF-12 comprises 12 items which can be aggregated into physical (PCS) and mental (MCS) component scores. The component scores were scored using RAND software and ranged from 0 to 100 (44). Lower scores on the PCS or MCS respectively represent a higher likelihood and severity of physical and mental health conditions. We defined low physical and mental quality of life as PCS≤50 and MCS≤42, respectively (44).

### Statistical Analyses

Descriptive statistics and statistical modelling were performed using IBM SPSS version 24 (45). Bootstrapping and internal validation was performed using R statistical software version 3.4.3 (46), using the “pROC” (47) and “rms” (48) packages, respectively.

We first determined the univariate association between each risk factor and risk of depression (HADS-D≥8). All risk factors which were significantly associated with risk of depression (p<0.05) were then entered into the multivariate logistic regression model, with risk of depression as the dependent variable. Forward and backward stepwise algorithms (p<0.10 for entry and removal) were run to select the final set of risk factors. The variance inflation factor values were calculated to detect multicollinearity between factors.

### Risk Score and Risk Stratification Development

A risk scoring system was created based on the multivariate logistic regression model to obtain a screening score. Each risk factor was assigned a score obtained by dividing its regression coefficient by the coefficient with the smallest value in the model, and rounded to the nearest integer. An individual's total risk score can then be computed by summing up the accumulated risk scores from the different risk factors. Based on the distribution of total scores, we stratified caregivers into three risk levels (i.e. low, intermediate and high). The relative risks of the intermediate and high-risk groups were calculated using the rate of HADS-D≥8 in the low risk group as reference.

### Performance of the Model

The discriminative ability of the risk scoring system was assessed by calculating the area under the receiver operating characteristic curve (AUC). Confidence intervals for the AUC was estimated using the method of Delong et al (49). The agreement between observed and predicted rates of caregivers at risk of depression was tested with the Hosmer-Lemeshow test. A p-value of <0.05 would mean that the model did not fit the data. The model was internally validated by bootstrapping 200 samples from the original data. The bootstrapping technique creates a large number of sample datasets by randomly sampling the original dataset with replacement sets to produce a less biased or an optimism adjusted estimate of the AUC (50).

To assess the extent to which adding all risk factors to the model will influence the discriminative ability, the AUC of the model with all risk factors were calculated. Overall χ2 tests were used to compare rates of “caregivers at risk of depression” by risk group.

Predictive validity was assessed by determining the tool's ability to predict 1) risk of depression, 2) low PCS and 3) low MCS, respectively, during the follow-up at 3-months post-discharge. We also tabulated the rates of caregivers with risk of depression, low PCS and low MCS at 3-months post-discharge.

## Results

### Demographic Characteristics

We recruited 274 caregiver-patient dyads. On admission, 92 (33.6%) caregivers were at risk of depression. At 3-months post-discharge, we excluded 72 caregivers (25 declined follow-ups primarily due to time constraints, 36 caregivers had care-recipients who passed away and 11 had care-recipients who were admitted into nursing homes). Amongst the 202 included caregivers at 3 months, 89 (44.1%) were at risk of depression. Descriptive statistics are shown in Table 1.

**Table 1:** Characteristics of caregivers and care-recipients (N = 274)

**Caregiver Characteristics**	N (%)*
Age in years, mean ± SD	59 ± 10.5
Sex (Female)	178 (65)
Single marital status	108 (39.4)
Low education (secondary and lower)	175 (63.9)
Ethnicity	
Chinese	229 (83.6)
Malay	20 (7.3)
Indian	16 (5.8)
Others	9 (3.3)
Working (full/ part-time)	136 (49.6)
Relationship to care-recipient	
Spouse	47 (17.2)
Child	194 (70.8)
Others	33 (12)
Living with care-recipient	232 (84.7)
Presence of live-in domestic helper	135 (49.3)
Years of caregiving	
≤5 y	149 (54.4)
&gt;5 to ≤10 y	103 (37.6)
&gt;10 y	22 (8)
Mastery (range 0 to 21), mean ±SD	8.58 ± 3.3
ZBI Burden (range 0 to 16), mean ±SD	6.05 ± 3.8
Depression, HADS subscale (range 0 to 21), mean ±SD	5.91 ± 4.5
**Care Recipient Characteristics**	N (%)*
Age, year, mean ±SD	85.3 ± 8
Female	175 (63.9)
Dementia diagnosis	138 (50.4)
Barthel Index scores (range 10 to 30), mean ±SD	19.5 ± 5.6
NPI-Q Severity (range 0 to 36), mean ±SD	7.37 ± 6.6


*N (%) unless otherwise indicated; HADS, Hospital Anxiety and Depression Scale; NPI-Q, Neuropsychiatric Inventory Questionnaire; ZBI, Zarit Burden Interview

### Model Development

The 11 caregiver risk factor variables identified are shown in Table 2. Based on univariate analysis, three factors were significant and therefore eligible for the multivariate model: low mastery (OR: 9.27, 95% CI: 5.22–16.47), high caregiver burden (OR: 6.38, 95% CI: 3.67–11.12) and low education (OR: 2.51, 95% CI: 1.23–3.75). The variance inflation factor values (low mastery =1.21, high caregiver burden =1.21, and low education =1.02) confirmed no evidence of multicollinearity between these predictors.

**Table 2:** Unadjusted associations between risk factors and risk of depression on admission

**Risk Factor**	**Odds Ratio (95% CI)**	**p value**
**Background and contextual factors**		
Single marital status	1.13 (0.67–1.88)	0.65
Spouse of care-recipient	1.60 (0.84–3.03)	0.15
Living with care-recipient	1.32 (0.64–2.70)	0.46
Low education (secondary and lower)	2.51 (1.23–3.75)	0.007
Age of caregiver	1.26 (0.74–2.15)	0.39
Public housing	1.77 (0.89–3.49)	0.102
Working (full/part-time)	1.24 (0.75–2.06)	0.39
No domestic helper	1.42 (0.86–2.35)	0.17
Female sex	1.02 (0.60–1.72)	0.95
**Psychological factors**		
Low mastery (scores ≥10)	9.27 (5.22–16.47)	&lt;.001
High burden (scores ≥8)	6.38 (3.67–11.12)	&lt;.001

### Model Performance

The final model containing three variables had an AUC of 0.82 (95% CI: 0.77–0.87). Internal validation with 200 bootstrap samples yielded an optimism-adjusted AUC of 0.82. The AUC of the final model is similar to the full model with all 11 caregiver risk factors (AUC: 0.83, 95% CI: 0.78–0.88). The Hosmer-Lemeshow test showed that the final model had no evidence of poor calibration (p = 0.72). Using the coefficient values from binary logistic regression, we assigned points to each of the risk factors (Table 3). The resultant risk scoring system had good discriminatory ability (AUC: 0.82, 95% CI: 0.77–0.87) which is similar to the original logistic regression model.

**Table 3:** Logistic regression and assigned points of risk factors

**Risk Factor**	**B**	**Odds Ratio (95% Confidence Interval)**	**p value**	**Points**^**A**^
Low mastery*	1.81	6.09 (3.30–11.24)	&lt;.001	3
High burden#	1.31	3.69 (1.99–6.85)	&lt;.001	2
Low education	0.60	1.82 (0.95–3.48)	0.067	1


Cutoff points: *Low mastery ≥10; #High burden ≥8; ∧Calculated by dividing regression coefficient with 0.60 (lowest value of regression coefficient obtained from model)

Using the total risk scores, we created three risk levels: low-risk (0 to 1 points), intermediate-risk (2 to 4), and high-risk (5 to 6). Rates of caregivers at risk of depression were 10.7%, 44.6%, and 73.3%, in the low, intermediate, and high-risk groups respectively (χ2=79.36, p< .001). Compared with the low-risk group, this represented increased relative risk of around four- and seven-fold in the intermediate and high-risk groups, respectively (Table 4).

**Table 4:** Risk of depression by risk group at index admission

**Risk group (scores)**	**Risk of depression in each risk group**, n/N (%)*	**Relative risk (95%CI)**
**On Admission**		
Low (0–1)	15/140 (10.7%)	1 (reference)
Intermediate (2–4)	33/74 (44.6%)	4.16 (2.42 to 7.15)
High (5–)	44/60 (73.3%)	6.84 (4.14 to 11.30)


Calculated by dividing number of caregivers at risk of depression (HADS-D ≥ 8) in each low-, intermediate, high-risk group by the total number of caregivers in that risk group.

### Predictive validity

At follow-up 3 months post-discharge, the AUCs of the risk scoring system for risk of depression (AUC: 0.72, 95% CI: 0.65–0.79), low PCS (AUC: 0.71, 95% CI: 0.64–0.79) and low MCS (AUC: 0.62, 95% CI: 0.58–0.74), were within acceptable range (51). Rates of risk of depression, low PCS and low MCS, increased across the three risk levels (p<0.001). Using the low-risk group as reference, this corresponded to relative risk of 2.07, 2.03 and 1.43, respectively, for the intermediate risk group; and 2.72, 2.48 and 1.67, respectively, for the high risk group (Table 5).

**Table 5:** Risk of depression, low MCS and low PCS by risk group at 3 months post discharge

**Risk group (Scores)**	**HADS-D (≥8)**	**Relative risk (95%CI)**	**MCS (≤42)**	**Relative risk (95%CI)**	**PCS (≤50)**	**Relative risk (95%CI)**
**3 Months Post Discharge**						
Low (0–1)	29/107 (27.1%)	1 (reference)	25/107 (23.4%)	1 (reference)	59/107 (55.1%)	1 (reference)
Intermediate (2–4)	32/57 (56.1%)	2.07 (1.41 to 3.05)	27/57 (47.4%)	2.03 (1.31 to 3.14)	45/57 (78.9%)	1.43 (1.15 to 1.78)
High (5–6)	28/38 (73.7%)	2.72 (1.89 to 3.91)	22/38 (57.9%)	2.48 (1.60 to 3.84)	35/38 (92.1%)	1.67 (1.37 to 2.03)


HADS-D, Hospital Anxiety and Depression Scale; MCS, mental component scores; PCS, physical component scores

## Discussion

In this study, we report the development of a caregiver-centric risk score to stratify caregivers of older adults with an unplanned admission into high-, intermediate- and low-risk groups for risk of depression. The final model consisted of educational level, burden and mastery, representing the ‘background and contextual factors', ‘secondary appraisal' and ‘mediator' domains in the stress-appraisal model (22). While the influence of caregiver burden and low education on caregivers' outcomes is consistent with past studies (34, 36), our findings on the prognostic potential of mastery on caregivers' risk of depression are novel. Notably, among the three components, mastery contributed the highest to the overall risk score. To our knowledge this is the first caregiver-centric screening tool that integrated validated mediating protective factors such as mastery with risk factors to identify caregivers at risk of depression. Our findings underscore the importance of evaluating mastery as an additional dimension in caregiver risk assessment for depression.

Using the risk score, intermediate and high-risk groups had increased risk of depression of approximately four- and seven-fold respectively compared with the low-risk group at admission. These findings highlight the need to provide more immediate and intensified interventions to those with intermediate and high risk groups. At 3-months post-discharge, intermediate and high risk groups still remained at two- to three-fold increased risk of depression compared to the low risk group, supporting the predictive validity of the risk stratification. Predictive validity is further supported by the increased risk of impaired quality of life in the intermediate to high-risk groups at 3-months post-discharge. The higher magnitude of risk in the mental compared with physical component of the quality of life, lends credence that it is the risk of depression that is driving the impaired quality of life.

A closer examination of the three risk levels reveals that relative to the low risk group, the intermediate risk group comprises caregivers with either low mastery or high burden whereas the high risk group has both low mastery and high burden. The differential relative risks in risk of depression on admission and at 3-months post-discharge between intermediate and high-risk groups corroborate the critical importance of screening both risk and protective factors in caregiver evaluation for risk of depression. With regards to the variable of educational level, it is listed as a resource in the caregiver appraisal model that may be drawn upon in coping with stress by mitigating the impact of the caregiving demand. It provides useful insights about the background and contextual factors that can inform the planning of individualized interventions such as caregiver education and coping strategy.

Taken together, the use of the caregiver screening tool is in line with the recent World Health Organization guidelines for integrated care for older adults, which strongly recommended needs assessment for family caregivers experiencing stress (52). Changes in healthcare with a shift from inpatients to community settings for chronic diseases, have placed increasing demands on family caregivers as they care for more complex, frail and chronically ill loved-ones. Family caregivers' well-being has wide healthcare, societal and financial implications (53). The incorporation of a caregiver-centric screening tool such as ours, leverages upon the window of opportunity the hospitalisation episode provides. During this period, the care team can identify vulnerable caregivers as part of routine evaluation and provide needed interventions while their loved ones are receiving medical care during the hospitalization. The adoption of such a tool would also encourage healthcare professionals to look beyond the needs of the patients to include the needs of caregivers, and challenge the healthcare system to be transformed towards one that provides training beyond traditional caregiver skills training to include areas such as self-care management.

A possible workflow is described here: Upon admission, all caregivers are given the caregiver screening tool to complete. The nurse will then tabulate the scores and determine the type of interventions and their urgency based on the risk accorded to the caregiver. High-risk caregivers could receive individualized counselling by social workers to further assess their specific caregiving needs during the hospitalisation period and where necessary, refer these individuals to caregiver interventional programs aimed at building caregiving skills and psychological resilience. Referrals to community respite care services can also be planned. In comparison, caregivers in the intermediate- and low-risk groups may not need as much assistance. Instead of individualized interventions, nurse-led caregiver education supplemented with educational materials could be offered. Lastly, it is important to follow-up on the high and intermediate groups, given that we found elevated longitudinal risk in both groups.

We would like to highlight some limitations in this study. The screening tool was developed from family caregivers of older adults hospitalized in a large Singapore hospital. Caution should be exercised when generalizing the findings to outpatient settings and other socio-cultural contexts. The use of this screening tool would be most suitable in populations that resemble our study population as possible differences with respect to culture and somatic symptoms may affect the detection of depression (54). Additionally, the screening tool is meant to serve as a signpost to direct clinicians to caregivers of various risk levels. A needs assessment should follow to better target individualized caregiver management from a multidisciplinary team. Not withstanding the inherent limitations of self-reported screening tools, this is outweighed by the potential benefit of routine screening on admission to enhance the detection of at-risk caregivers compared to the absence of such a tool.

We believe that our study paves the way for more future research into the use of caregiver-centric screening tools that evaluate both risk and protective factors for early detection and targeted intervention. To ensure that the tool is robust for both researchers and clinicians, we recommend that future work be directed towards further validation of this tool in a prospective sample with longer follow-up in other study populations. It would also be useful to validate the screening tool in populations which are culturally different, in non-acute care settings such as outpatient clinics, or in other caregiver populations. Future research could also explore the cost-benefit of these screening tools and how best they could be used in the clinical settings. Studies could also assess the effectiveness of using structured risk stratification tools to detect vulnerable caregivers.

## Conclusion

The caregiver-centric screening tool is a novel, 12-item, self-administered tool premised on the stress-appraisal model that can identify caregivers of hospitalized older adults who are at risk of depression on admission and has predictive validity for risk of depression and impaired quality of life at 3-months post-discharge. Coupled with its relatively low respondent burden, it can be incorporated as part of routine evaluation upon hospitalization to enable early identification of at-risk caregivers. The provision of risk stratification scores derived from psychological “protective” and “risk” constructs of mastery and burden enhances its utility as holistic screening tool, so that appropriate and prompt intervention can be rendered to at-risk caregivers.

## Electronic supplementary material

Appendix 1: Caregiver Screening Tool
