# Supplementary material for: Development of a Brief Caregiver-centric Screening Tool to Identify Risk of Depression among Caregivers of Hospitalized Older Adults
Source: J Nutr Health Aging. 2019 Apr 25;23(6):578–85. doi: 10.1007/s12603-019-1197-7 (PMC6586909; doi:10.1007/s12603-019-1197-7)
Supplement: Supplementary file 1 — Appendix 1: Caregiver Screening Tool [file mmc1.pdf]

Appendix 1: Caregiver Screening Tool

Caregiver-centric Screening Tool

The caregiver-centric screening tool consists of 12-items including two psychological constructs, mastery and burden. Scoring instructions and risk stratifications are provided to calculate a caregiver's score and risk of depression.

COPYRIGHT

Appendix 1: Caregiver Screening Tool

Please check the appropriate box.

1 Highest level of education

- ☐ Tertiary level education
- ☐ Secondary level education
- ☐ Primary level/ no formal education

2 The questions below reflect how you sometimes feel when you are taking care of your care recipient. Please check the appropriate box.*

		Never	Rarely	Sometimes	Quite Frequently	Nearly Always	
2.1	Do you feel that because of the time you spend with your care recipient that you don't have enough time for yourself?	☐	☐	☐	☐	☐	
2.2	Do you feel stressed between caring for your care recipient and trying to meet other responsibilities for your family or work?	☐	☐	☐	☐	☐	
2.3	Do you feel strained when you are around your care recipient?	☐	☐	☐	☐	☐	
2.4	Do you feel uncertain about what to do about your care recipient?	☐	☐	☐	☐	☐	
						Total	

*Scoring:
Never = 0, Rarely = 1, Sometimes = 2, Quite Frequently = 3, Nearly Always = 4
High Burden: Total score ≥ 8

COPYRIGHT

Appendix 1: Caregiver Screening Tool

3 The following questions are about how much control you have over your life in general (including control over your personal time, family life, social life and work). Please check whether you strongly disagree, disagree, agree or strongly agree with each of these statements.*

		Strongly Disagree	Disagree	Agree	Strongly Agree	
3.1	I have little control over the things that happen to me.	☐	☐	☐	☐	
3.2	There really is no way I can solve some of the problems I have.	☐	☐	☐	☐	
3.3	I can do just about anything I really set my mind to do.	☐	☐	☐	☐	
3.4	Often I feel helpless in dealing with the problems of life.	☐	☐	☐	☐	
3.5	Sometimes I feel like I am being pushed around in life.	☐	☐	☐	☐	
3.6	What happens to me in the future mostly depends on me.	☐	☐	☐	☐	
3.7	There is little I can do to change many of the important things in my life.	☐	☐	☐	☐	
					Total	

*Scoring:
Scoring for 3.1, 3.2, 3.4, 3.5: Strongly Disagree = 0, Disagree = 1, Agree = 2, Strongly Agree = 3
Scoring for 3.3, 3.6 : Strongly Disagree = 3, Disagree = 2, Agree = 1, Strongly Agree = 0
Low Mastery: Total score ≥10

COPYRIGHT

Appendix 1: Caregiver Screening Tool

Individual Item Score

No.	Risk Factor	Scoring Criteria	Score
1	Highest Education Level		
	Low (Secondary and below)	0	
	High (Tertiary)	1	
2	Burden		
	Low Burden (0-7)	0	
	High Burden (8-16)	2	
3	Mastery		
	High Mastery (0-9)	0	
	Low Mastery (10-21)	3	
		**Total Score**	
		**Risk Level***	

Risk Level*

Total Score	Risk Level
0 – 1	Low
2 – 4	Intermediate
5 – 6	High

COPYRIGHT

Copyright - To be reproduced with Authors' permission
